# The Common Task

**Published:** 1908-02-22

**Authors:** 


### Nursing Administration.

### THE COMMON TASK.

### Cost of Food in Workhouses.

The low cost of food in some provincial workhouses is matter for admiring comment on the part of those who have abundant opportunity for examining into the quality of the rations provided. The average weekly cost per head in Cornwall, Devon, and Somerset nowhere exceeds 3s., and in ten workhouses is less than 2s. 6d. Mr. Preston Thomas, the District Inspector, is astonished to see what excellent meals can be provided for this amount, and he draws attention to the fact that prices vary much, even the quartern loaf showing a difference of 1¾d. in price as between Redruth and Tavistock, so that this economical result cannot be said to depend entirely on cheap supplies. The cost of the inmates' food in certain carefully administered workhouses in London is about the same, averaging at Wandsworth, for instance, almost exactly 2s. 6d. a week. It is exceedingly interesting to find that bare maintenance sufficient to keep in good health persons of varying ages, many of whom are engaged in active work, can be procured in town and country for so small a sum. It could not be contended that this amount represents a fair average expenditure for wage-earners, but it unquestionably suffices for the requirements of health and is the foundation on which more elaborate diets are built up. If we reckon that a sum of about 1s. a head per week is saved by dealing with large quantities of provisions (an assumption which we consider doubtful), then it appears that an allowance of 6d. a day should suffice to keep an ordinary person in good health, excluding anything in the way of luxuries, but admitting a certain proportion of sweet things and meat. This fact has a distinct bearing on the old-age pension question, and no housekeeper for large numbers should omit to bear it in mind.

### Primary Nursing Technique for First-Year Pupil Nurses.

This useful manual by Isabel McIsaac (Macmillan and Co., New York) is admirably fitted for the use of probationers during their first year, and will save much bewilderment to the pupils who never clearly understand until they have seen directions in print. The practical chapters on giving baths, on bed-making, on symptoms, and on the administration of medicines are excellent. Those on ward routine are less likely to be useful to Englishwomen, the procedure varying greatly in American institutions from what is usual in this country. The merit of the book lies in its admirable arrangement. If to instruct is, as the Latin grammar declares, "to draw up in order," then these pages should stand high among manuals for pupil nurses. The writer justly asserts "It would require a very unusual mind to set in order and assimilate the masses of unrelated information which so often constitute the nurse's instruction. Much needs to be done and easily might be done to bring the theoretical and practical into proper relation. Too often they are separated by so sharp a line that theory seems suspended in mid-air while practice struggles awkwardly and inefficiently alone." We specially commend her suggestion that printed or type-written directions should be posted for reference by the nurses for the preparation of patients for operation. "Nurses should not be taxed with memorising any great amount of such detail."

